# Traditional serrated adenomas of the upper digestive tract

**DOI:** 10.1136/jclinpath-2015-203258

**Published:** 2015-10-14

**Authors:** CA Rubio

**Keywords:** GASTRIC CANCER, PANCREATIC CANCER, GALL BLADDER CANCER, OESOPHAGUS

## Abstract

For many years, it was generally accepted that the vast majority of the colorectal carcinomas (CRCs) evolved from conventional adenomas, via the adenoma–carcinoma sequence. More recently, serrated colorectal polyps (hyperplastic polyps, sessile serrated polyps and traditional serrated adenomas (TSAs)) have emerged as an alternative pathway of colorectal carcinogenesis. It has been estimated that about 30% of the CRC progress via the serrated pathway. Recently, TSAs were also detected in the upper digestive tract. In this work, we review the literature on TSA in the oesophagus, the stomach, the duodenum, the pancreatic main duct and the gallbladder. The review indicated that 53.4% (n=39) out of the 73 TSA of the upper digestive tract now in record showed a simultaneously growing invasive carcinoma. As a corollary, TSAs of the upper digestive tract are aggressive adenomas that should be radically excised, either endoscopically or surgically, to rule out the possibility of a synchronously growing invasive adenocarcinoma or to prevent cancer progression. The present findings substantiate a TSA pathway of carcinogenesis in the upper digestive tract.

## Introduction

Up to 1990, it was generally accepted that the vast majority of the colorectal carcinomas (CRCs), the most common carcinoma in the lower digestive tract, evolved from foci of dysplastic mucosa, referred to as conventional colorectal adenomas, via *the adenoma–carcinoma sequence.*^1^ In this paradigm, hyperplastic polyps (HPs) were considered innocuous.

In 1990, Longacre and Fenoglio-Preisser described the serrated adenoma, a novel adenoma phenotype typified by villous-like elongations displaying unlocked serrations lined with dysplastic cells.^2^ In that work, 37% of 110 mixed hyperplastic adenomatous polyps/serrated adenomas contained foci of significant dysplasia and 11%, areas of intramucosal carcinoma. In 2001, Jass postulated that the adenoma–carcinoma sequence might not apply to all CRC and that the serrated pathway comprising HP, mixed polyps and serrated adenomas could be the missing link.^3^ In later years, the *serrated pathway* of colorectal carcinogenesis in the lower digestive tract has received international acceptance.^4^ ^5^ In this model, colorectal HP, sessile serrated adenoma/polyps (SSA/P) and traditional serrated adenomas (TSAs) are regarded early histological potential precursors of colorectal serrated carcinomas.^6^ ^7^

In a recent work, we found two distinct phenotypes of TSA in the colon and rectum in Iceland;^8^ one was typified by unlocked serrated crypts (US-TSA) as described by Longacre and Fenoglio-Preisser^7^ and the other by microtubular dysplastic structures,^9^ also called ectopic crypt formations (ECFs).^10^ These findings were in concert with those of Kim *et al*^11^ showing that only 79% of TSAs had ECFs, with those of Wiland *et al*^12^ showing that only 62% of the TSAs had ECFs, and those of O'Brien *et al*^6^ postulating that ECFs were related to villous morphology/architecture rather than to serrated.

More recently, TSA were found in the oesophagus,^13^ the stomach,^14^ the duodenum,^15^ the pancreas,^16^ and the gallbladder.^17^ Taking into account the present worldwide concern for that adenoma phenotype in the lower digestive tract (colon, rectum^18^ and appendix^19^), it was consider of interest to review the published literature on TSA found in the upper digestive tract.

In this review, the upper digestive tract includes the oesophagus, the stomach, the duodenum, the liver including the biliary tract and the pancreas.^20^

### TSA of the oesophagus

**Figure 1:**
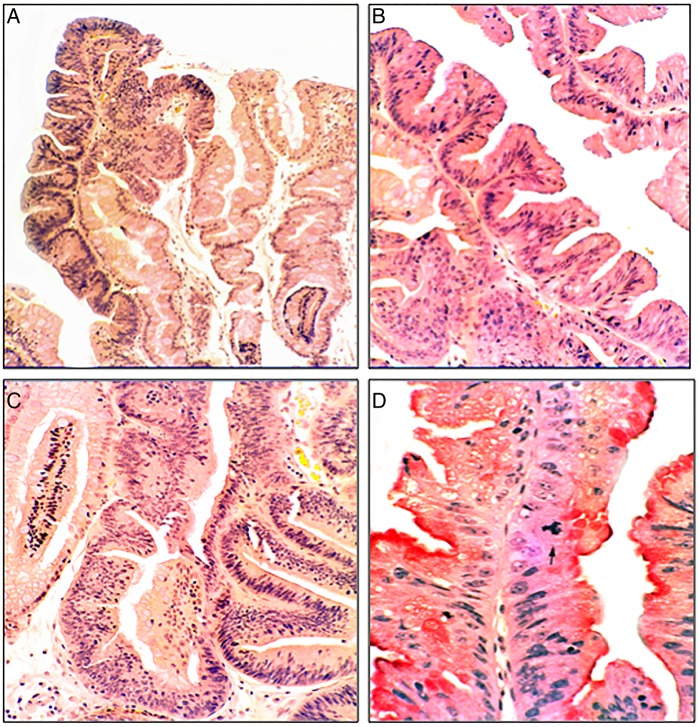
Traditional serrated adenoma (TSA) of Barrett’s oesophagus. (A) Low-power view of the TSA (H&E, ×4). (B) Closer view showing unlocked serrated configurations with low-grade dysplasia (H&E, ×10). (C) Basal aspect of the TSA with high-grade dysplasia (H&E ×20). (D) Detail from another area of the TSA of the oesophagus showing a tripolar mitosis (periodic acid Schiff stain, ×40).

The first case of TSA of the oesophagus was reported in 2013.^13^ It was found at the margin of an adenocarcinoma in a patient with Barrett’s oesophagus. At histology, the lesion showed epithelial outgrowths with unlocked serrated crypts lined with high-grade dysplasia, atypical mitoses (figure 1) and high cell proliferation (Ki-67). The Barrett’s mucosa exhibited non-dysplastic glands with intestinal metaplasia.

A search for cases of adenomas of the oesophagus in the database of this Department (1994–2014) yielded 11 adenomas; one was a TSA.^13^ No other case of TSA of the oesophagus has been reported in the literature.

### TSA of the stomach

**Figure 2:**
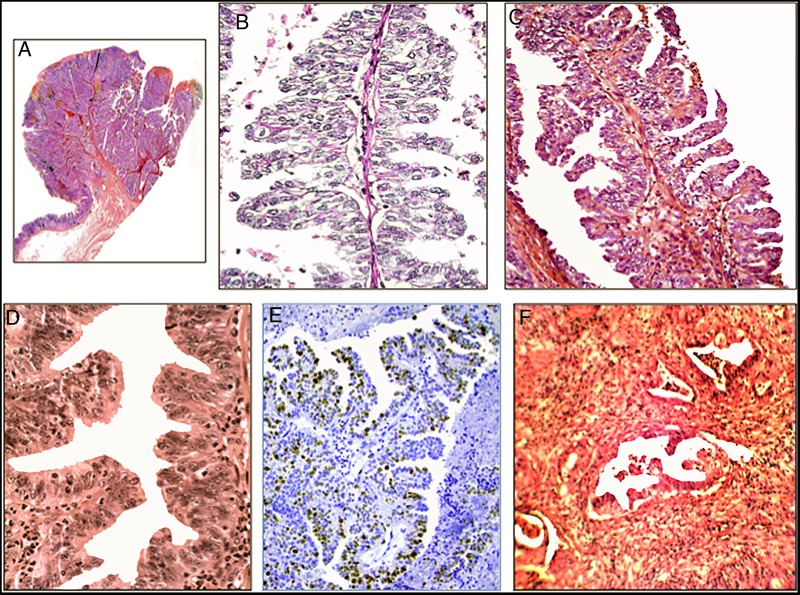
Traditional serrated adenoma (TSA) of the stomach. (A) Section from the resected specimen (H&E, ×1). (B) ‘Christmas-tree’-like serrated configuration (H&E, ×10). (C) Another area with ‘Christmas-tree’-like serrated configuration with high-grade dysplasia (H&E, ×10). (D) Detail from TSA showing unlocked serrated configurations with high-grade dysplasia (H&E, ×20). (E) ‘Christmas-tree’-like serrated configuration showing cell proliferation (Ki67, batch MIB1, ×10). (F) Invasive carcinoma arising in a gastric TSA (periodic acid Schiff stain (PAS), ×4).

The first case of TSA of the stomach was reported in 2001.^14^ The lesion was epitomised by villous-like protrusions lined with unlocked saw tooth-like crypts, lined with dysplastic cells (figure 2). Subsequently, new cases of TSA were reported from this Hospital^21–24^ as well as from other hospitals in disparate countries such as Tunisia,^25^ Japan,^26^ Turkey^27^ and South Korea.^28^ More recently, Dr H Szabo from Hungary consulted us for a gastric adenoma in a Hungarian patient. The lesion was a TSA exhibiting high-grade dysplasia and invasive carcinoma.

Notably, out of the 35 gastric TSA recorded, 74.3% (n=26) exhibited invasive carcinoma (table 1). As a comparison, only 15% of the conventional tubular or villous adenomas of the stomach reported in the literature showed invasive growth.^29^

**Table 1:** The number of traditional serrated adenomas (TSAs) of the upper digestive tract reported in the literature, having high-grade dysplasia or invasive carcinoma

References	TSAs with invasive carcinoma	Total no. of TSA
Oesophagus^13^	1	1
Stomach^14^ ^21–28^	26	35
Duodenum^15^ ^30–32^	10	35
Pancreas^16^	1	1
Gallbladder^17^	1	1
All, n (%)	39 (53.4%)	73 (100%)

A search for cases of adenomas of the stomach in the database of this Department (1994–2014) yielded 153 adenomas. Of these, 10 were TSA; one of them was recently found (unpublished).

### TSA of the duodenum

**Figure 3:**
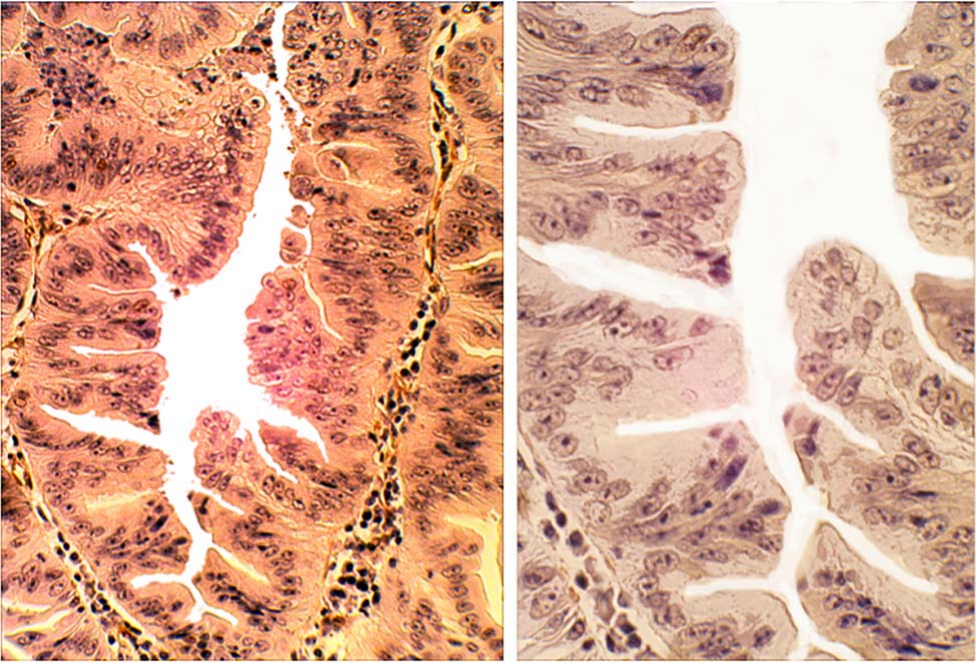
Traditional serrated adenoma (TSA) of the duodenum. (A) Detail of the TSA of the duodenum to show unlocked serrated crypts with high-grade dysplasia (H&E, ×10). (B) Another area of the TSA showing unlocked serrated configurations (H&E, ×20).

The first case of TSA of the duodenum was reported in 2004.^15^ The patient was a 78-year-old male with familial adenomatous polyposis. Eight years after colectomy, he developed a silent jaundice. A cholangiography showed a 2 cm long stricture in the distal choledocus. The surgical specimen showed a papillary tumour juxtaposing the papilla of Vater. Histology disclosed an adenomatous growth with unlocked saw tooth-like glands with high-grade dysplasia (figure 3). No invasive carcinoma was found. Six years later, a resected polyp in the ileostomy revealed a radically excised TSA with high-grade dysplasia. The patient is well up to this date. Following that original publication, 35 additional cases of TSA of the duodenum appeared in the literature;^30–32^ 28.6% (n=10) of the 35 cases showed invasive growth (table 1).

A search for cases of adenomas of the duodenum in the database of this Department (1994–2014) yielded 703 adenomas. Of these, five cases were TSA of the duodenum.

### TSA of the main pancreatic duct

**Figure 4:**
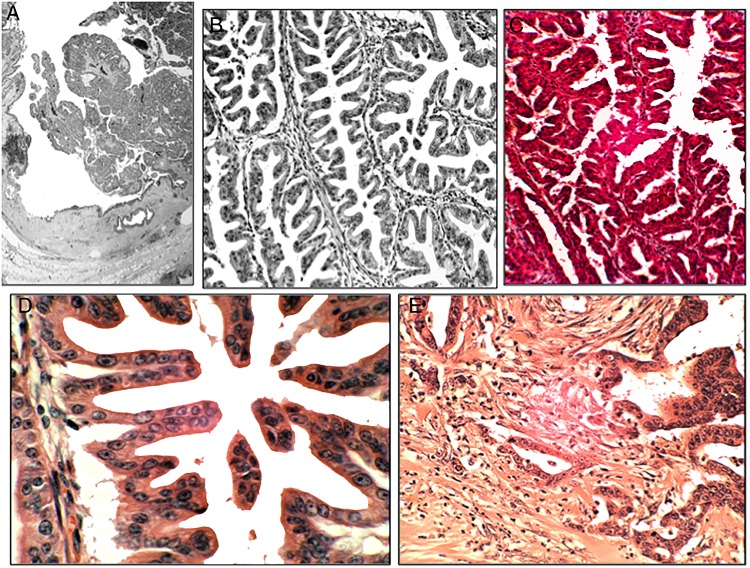
Traditional serrated adenoma (TSA) of the main pancreatic duct. (A) TSA of the main pancreatic duct (section from the resected specimen (H&E, ×1). (B) TSA showing unlocked serrated configurations lined with low-grade dysplasia (H&E, ×10). (C) Another area of the TSA showing eosinophilic cytoplasm (H&E, ×10. (D) Detail from the TSA of the main pancreatic duct, showing unlocked serrated configurations lined with low-grade dysplasia. Note the eosinophic cytoplasm (H&E, ×20). (E) Invasive carcinoma, arising in a TSA of the main pancreatic duct (H&E, 20×).

In 2005, we reported the first case of intraductal TSA of the pancreas in a 48-year-old male.^16^ The patient consulted for symptoms compatible with transient ischaemic attack. A CT scan revealed an irregular, lobulated lesion in the head of the pancreas. Histology revealed a tumour occupying the main pancreatic duct, built with unlocked saw tooth-like fronds lined with high-grade dysplasia. Invasive adenocarcinoma was found in the surrounding pancreatic tissue (figure 4).

A search for cases of adenomas of the pancreatic duct in the database of this Department (1994–2014) yielded five adenomas. Of these, one was a TSA.^16^ No other case of TSA of the main pancreatic duct has been reported in the literature.

### TSA of the gallbladder

**Figure 5:**
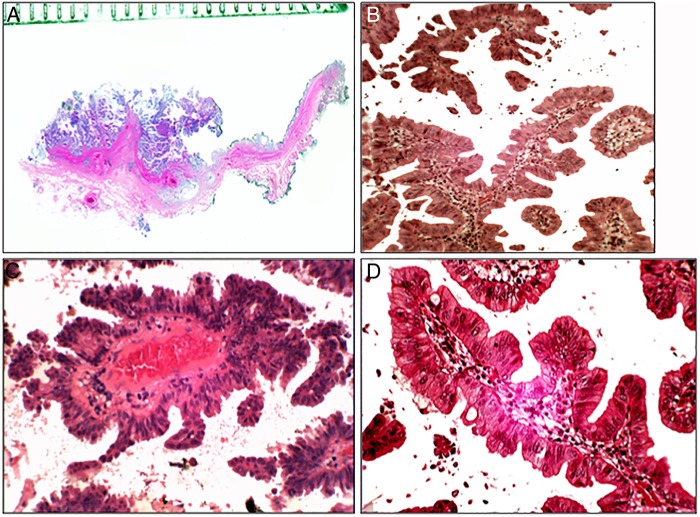
Traditional serrated adenoma (TSA) of the gall bladder. (A) TSA of the gallbladder (section from the resected specimen (H&E, ×1). (B) Low-power view of the TSA showing unlocked serrated configurations (H&E, ×4). (C) Closer view of the TSA showing unlocked serrated configurations with high-grade dysplasia (H&E, ×20). (D) Detail from another area of the TSA showing unlocked serrated configurations (H&E, ×20).

The first case of TSA in the gallbladder was reported recently.^17^ The patient, a 75-year-old male, has been treated for Crohn’s colitis since 1982. In 2002, he presented with right upper quadrant pain. A CT scan suggested primary gallbladder malignancy. Histology from the cholecystectomy revealed a moderately differentiated carcinoma engaging the middle part of the gallbladder. In the fundic region, a polypoid lesion showed unlocked serrations lined with high-grade dysplastic epithelium (figure 5). The subjacent *lamina propria*, muscularis *mucosae* and submucosa lengths the entire papillary adenoma was replaced by a series of sclerotic desmoplastic, branched hubs with low cellularity. The thriving serrated adenoma and the collection of minor desmoplastic stromal hubs were regarded as papillary at low-power microscopy. Since the adenocarcinoma showed no remnant adenoma, it was impossible to assess whether the carcinoma had originated in a similar serrated adenoma as that in the fundus.

A search for cases of adenomas of the gallbladder in the database of this Department (19940101-20141231) yielded seven adenomas. Of these, one was a TSA.^17^

No other case of TSA of the gall bladder has been reported in the literature.

## Discussion

The biological significance of serrated polyps in the lower digestive tract, including TSAs, has attracted much interest in later years. It has been estimated that about 30% of the CRCs evolve via the serrated pathway.^6^

In the upper digestive tract, conventional adenomas are the most frequent histological phenotypes. This review showed that TSAs also occur in the oesophagus, the stomach, the duodenum, the main pancreatic duct and the gallbladder, albeit to a much lower extent.

All TSAs in upper digestive tract so far reported displayed unlocked serrated structures (US-TSA). TSAs with microtubular dysplastic structures,^9^ ^33^ currently known as TSAs with ECF^10^ have not been yet found in the upper digestive tract.

Table 1 shows that of the 73 cases of TSA tract so far reported in the literature 53.4% (n=39) had invasive carcinoma. Although the cause(s) for this aggressive behaviour remains elusive, it would appear that not only the degree of cellular severity, but also the histological configuration (ie, with unlocked serrations) might have play a particular role in their virulence. This assumption is not surprising, considering that it has repeatedly been demonstrated that the presence of villous configurations in colorectal adenomas increases the frequency of detecting a synchronously growing invasive carcinoma.^34^

TSAs of the upper digestive tract are aggressive adenomas that should be radically excised, either endoscopically or surgically, to rule out the possibility of a synchronously growing invasive adenocarcinoma or to prevent cancer progression.

The present findings substantiate a TSA *pathway* of carcinogenesis in the upper digestive tract.

**Take home messages**

- In later years serrated colorectal polyps, including traditional serrated adenomas (TSA), have emerged as an alternative pathway of colorectal carcinogenesis.
- Recently, TSAs were also detected in the esophagus, the stomach, the duodenum, the pancreatic main duct and the gallbladder.
- Out of the 73 TSA of the upper digestive in record, 53.4% (n=39) showed a simultaneously growing invasive carcinoma.
- TSAs of the upper digestive tract are aggressive adenomas that should be radically excised, either endoscopically or surgically to rule out the possibility of a synchronously growing invasive adenocarcinoma or to prevent cancer progression.
- The present findings substantiate a TSA pathway of carcinogenesis in the upper digestive tract.
